# Metabolic Profiling of Female Tg2576 Mouse Brains Provides Novel Evidence Supporting Intranasal Low-Dose Pioglitazone for Long-Term Treatment at an Early Stage of Alzheimer’s Disease

**DOI:** 10.3390/biomedicines8120589

**Published:** 2020-12-09

**Authors:** Ling Rong Wong, Peiyan Wong, Paul Chi-Lui Ho

**Affiliations:** 1 Department of Pharmacy, Faculty of Science, National University of Singapore, Singapore 117543, Singapore; phawlr@nus.edu.sg; 2 Neuroscience Phenotyping Core, Department of Pharmacology, Yong Loo Lin School of Medicine, National University of Singapore, Singapore 117456, Singapore; phcwp@nus.edu.sg; 3 Neuroscience and Behavioral Disorders Programme, Duke-NUS Medical School, Singapore 169857, Singapore

**Keywords:** Alzheimer’s disease, intranasal delivery, metabolic profiling, pioglitazone, PLGA nanoparticles

## Abstract

Accumulating evidence suggests that disruptions in brain energy metabolism may be a key player in the pathogenesis of Alzheimer’s disease (AD). Pioglitazone (PIO) has been found to exert beneficial effects on metabolic dysfunction in many AD preclinical studies. However, limited success in clinical trials remains an obstacle to its development for the treatment of AD. PIO’s poor brain penetration was often cited as a contributing factor to the lack of clinical benefit. In this study, we prepared PIO-loaded poly(lactic-co-glycolic acid) (PLGA) nanoparticles and administered them as suspended nanoparticles via nebulization. Preliminary investigation of drug distribution to the brain revealed comparatively reduced systemic exposure after administering PIO nanoparticles via the intranasal route. In vitro, extracellular flux analysis showed significantly raised spare respiratory capacity when cells were treated with low-dose PIO nanoparticles. Tg2576 transgenic mice treated with low-dose PIO nanoparticles over four months exhibited an overall trend of reduced hyperactivity in open field tests but did not show any visible effect on alternation rates in the Y-maze task. Subsequent ^1^H NMR-based metabolic profiling of their plasma and different brain regions revealed differences in metabolic profiles in the cerebellum, cortex, and hippocampus of Tg2576 mice after long-term PIO treatment, but not in their midbrain and plasma. In particular, the specificity of PIO’s treatment effects on perturbed amino acid metabolism was observed in the cortex of transgenic mice with increases in alanine and N-acetylaspartate levels, supporting the notion that PIO treatment exerts beneficial effects on impaired energy metabolism associated with AD. In conclusion, inhalation exposure to PIO nanoparticles presents an exciting opportunity that this drug could be administered intranasally at a much lower dose while achieving a sufficient level in the brain to elicit metabolic benefits at an early stage of AD but with reduced systemic exposure.

## 1. Introduction

Alzheimer’s disease (AD) is a progressive, irreversible neurodegenerative disorder that affects wide areas of the cerebral cortex and hippocampus [1], which are brain regions critical to learning and memory. Decades of research have revealed detailed information about the disease’s molecular pathogenetic events, yet the exact pathogenesis of AD remains elusive [2]. Although the disease is still defined by amyloid and tau protein abnormalities, many researchers are gradually transiting away from the simple assumption of linear causality that was proposed in the original amyloid cascade hypothesis as amyloid-lowering approaches have yet to translate to cognitive benefits in human clinical trials [3]. Increasing evidence suggests that metabolic alterations strongly influence the initiation and progression of AD [4]. This is anchored by positron emission tomography imaging studies revealing abnormalities in cerebral glucose metabolism in both familial and sporadic AD cases, including before the onset of cognitive decline [5]. This apparent decrease in the brain’s ability to metabolize glucose with disease progression suggests that improving the neuronal energy state at an early stage may impede the disease process and delay or prevent the undesirable progression of clinical manifestations. Indeed, type 2 diabetes mellitus, a metabolic disorder that is strongly associated with insulin resistance, has been implicated in AD with considerable overlap in risk factors, comorbidities, and pathophysiological mechanisms [6]. There is also a growing number of agents targeting metabolism and bioenergetics in the AD drug development pipeline [7].

Pioglitazone (PIO) is a thiazolidinedione drug that is commonly prescribed to manage type 2 diabetes mellitus [8]. Multiple preclinical studies have shown that PIO exerts therapeutic benefits on metabolic dysfunction in experimental models of AD. For example, cerebral blood flow and glucose uptake were significantly improved in AD mice after long-term oral treatment with PIO [9,10]. Short-term treatment with PIO restored mitochondrial enzyme complex activities in the hippocampus of rats injected with amyloid-β (Aβ) peptides [11]. A recent study also demonstrated the specificity of PIO’s treatment effects on metabolic alterations in the cortex and cerebellum of AD mice [12]. A pilot 6-month clinical trial in patients with mild AD and type 2 diabetes mellitus showed cognitive improvements after treatment with PIO, and this was accompanied by improvements in regional cerebral blood flow deficits in the parietal lobe [13]. Conversely, in another 18-month trial comprising non-diabetic patients with mild-to-moderate AD, no efficacy was demonstrated on clinical outcome measures after PIO treatment [14], suggesting that mild-to-moderate AD is likely not a suitable population for the study of PIO treatment. In 2013, a 5-year global phase 3 trial was initiated to study the efficacy of low-dose PIO to delay the onset of mild cognitive impairment due to AD in non-cognitively impaired elderly participants whose increased risk of AD was determined by a predictive biomarker [15]. Unfortunately, this trial was discontinued in early 2018 due to inadequate treatment effect observed [16]. The relatively low dose (0.8 mg) administered could be a contributing factor to the lack of clinical benefit because PIO was reported to demonstrate limited brain penetration [17,18]. Furthermore, PIO was found to be a substrate of P-glycoprotein, and this was proposed to contribute to the low drug exposure observed in the brain [18]. On the other hand, the use of a low dose is understandable as patients with long-term and high-dose exposure to PIO have been associated with an increased risk of bladder cancer [19], although current evidence concerning this association remains conflicting [20].

Given these circumstances, it would, therefore, be advantageous if PIO could be delivered to the brain in sufficient amounts to elicit treatment benefits but at the same time, with a lower risk of systemic exposure. Moreover, long-term administration of therapeutics starting at an early stage before the onset of substantial AD pathology would help achieve preventive or delaying effects. As such, a non-invasive approach to drug administration will be much more appealing to users if long-term use is necessary. In this aspect, we propose that the intranasal approach can serve as a non-invasive method to enable therapeutic agents to circumvent the blood-brain barrier (BBB), thus facilitating their direct delivery to the brain. Furthermore, this pathway effectively reduces systemic exposure and lowers the risk of side effects that may be associated with chronic drug usage [21]. Notably, the formulation of nanocarrier systems is considered to be a promising approach in nose-to-brain drug delivery [22]. In this study, we prepared PIO-loaded poly(lactic-co-glycolic acid) (PLGA) nanoparticles (NP) for intranasal application, and specifically, we attempted inhalation as an alternative approach to nasal instillation. Preliminary investigation of drug distribution to the brain revealed comparatively reduced systemic exposure after low-dose PIO NP inhalation. Additionally, we demonstrated that our PIO NP formulation enhances mitochondrial bioenergetics in an AD in vitro model. Open field tests performed on Tg2576 transgenic mice revealed that those treated with low-dose PIO NP via inhalation over 4 months exhibited a general trend of reduced hyperactivity, although the treatment did not have any visible effect on their alternation rates in the Y-maze task. Our metabolic profiling results are also the first to demonstrate that low-dose PIO NP administered via inhalation over the long term is able to affect energy metabolism in specific brain regions, thus presenting the exciting opportunity of employing a nose-to-brain drug delivery approach in the repurposing of PIO for improving the cerebral energy state at an early stage of AD.

## 2. Materials and Methods

### 2.1. Chemicals

Resomer RG 502 (PLGA) was obtained from Evonik (Darmstadt, Germany). D-α-Tocopherol polyethylene glycol succinate (TPGS, Sigma 57668), 3-(trimethylsilyl)propionic-2,2,3,3-d_4_ acid sodium salt (TSP-d_4_, Sigma 269913), and deuterium oxide (Sigma 151882) were purchased from Sigma-Aldrich (St. Louis, MO, USA). PIO was purchased from Cell Molecular Pharmaceutical R&D (Xi’an, China). Trehalose dihydrate (TCI T0331) was purchased from Tokyo Chemical Industry (Tokyo, Japan). All other reagents were of analytical grade and used as received.

### 2.2. Preparation of PIO-Loaded PLGA-TPGS Nanoparticles

PIO-loaded PLGA nanoparticles in this study were prepared by the single-emulsion method with vitamin E-TPGS as the emulsifying agent [23]. 50 mg PLGA and 1 mg PIO were first separately dispersed in a minimal amount of chloroform. The solubilized PIO was added into the PLGA solution, and the mixture was vortex-mixed to ensure that the drug was homogenously dispersed. This drug-polymer mixture was then carefully added dropwise into 1 mL 0.3% (*w*/*v*) TPGS solution contained in a 13 mm × 100 mm glass test tube held on a high vortex. The primary emulsion obtained was immediately homogenized in 10 s bursts using a 3 mm microtip probe sonicator (Model 500, Fisher Scientific, Pittsburgh, PA, USA). The homogenous white emulsion formed was emptied into a bulk stirring 0.3% (*w*/*v*) TPGS solution. The mixture was stirred vigorously for at least 4 h to allow the nanoparticles to harden as the organic solvent evaporated away. The translucent quality of the stirring solution should increase as particle size decreases during the solvent evaporation process. The hardened nanoparticles were collected by centrifugation at 18,000 *g* for 30 min at 20 °C and washed with Milli-Q water. Trehalose solution was added into the final pellet resuspension as a cryoprotectant and mixed well. The final product was rapidly frozen at −80 °C and lyophilized. Drug loading amounts in the nanoparticles were quantified using reversed-phase HPLC. Particle size and polydispersity index (PDI) of the nanoparticles were determined using the dynamic light scattering method (Zetasizer Nano-ZS90, Malvern Instruments, Worcestershire, UK). The Zeta potential of the nanoparticles was also measured using the same instrument. Morphology of the nanoparticles (without the addition of trehalose) was observed by field emission scanning electron microscopy (FESEM; JSM-6701F, JEOL, Peabody, MA, USA).

### 2.3. Inhalation Exposure to Aerosol Generated by Nebulization of PIO-Loaded PLGA-TPGS Nanoparticles

#### 2.3.1. Animals

Adult male C57BL/6 mice (10–12 weeks of age) were obtained from InVivos (Singapore). The mice were housed in groups (maximum of 5 mice per cage) under standard conditions of humidity, temperature, and 12 h light/dark cycle with ad libitum access to food and water. All mice were maintained under constant conditions for 4 days prior to experiments. All animal experiments were conducted in accordance with the Singapore National Advisory Committee on Laboratory Animal Research (NACLAR) guidelines and approved by the Institutional Animal Care and Use Committee (IACUC), National University of Singapore (IACUC Protocol # R17-0064, approved on 22 February 2017).

#### 2.3.2. Aerosol Generation System for Mouse Inhalation

The aerosol generation system for mouse inhalation used in our study was adapted with modifications from a previous design [24]. Briefly, 2 holes were cut at the sides of a leakproof plastic container (15 cm × 8 cm × 7 cm) for connecting the inhalation exposure tubes (EMMS, Bordon, UK) such that only the nose of each mouse would be exposed to the aerosol cloud, which was generated by nebulizing the dosing solution with a medical nebulizer (NE-C900, Omron, Japan). The interior of the container wall was lined with plastic mesh to prevent the animals from entering the container. Another hole was cut at the bottom of the container to interface the nebulizer’s medication tank via a plastic adapter. A waterproof plastic-compatible silicone sealant was applied to the edges of all cut holes to minimize leakage at these sites. Finally, a small hole was created in the container’s cover to allow for ventilation. The entire container, connected with the inhalation exposure tubes, was set on an acrylic stand to give the medication tank clearance below the container. This elevation also served to prevent the animals from escaping onto the surrounding BSC surface.

Mice were acclimated to restraint in the inhalation exposure tubes for one week prior to the actual experiment. Animals were trained to enter and remain in these tubes until released. During acclimation, mice were subjected to an aerosol generated by nebulizing 7 mL normal saline solution. Time spent by the animals in the inhalation exposure tubes was increased up to 30 min at a gradual rate over the entire training week. This training served to minimize confinement stress during the actual PIO NP aerosol exposure period.

#### 2.3.3. In-Vivo Comparative Study of Drug Distribution Profiles

Mice were split into 3 treatment groups: (A) PIO solution (5 mg/kg, i.n., nasal instillation), (B) PIO solution (15 mg/kg, p.o.) and (C) PIO-loaded PLGA-TPGS nanoparticles (PIO NP) (estimated 0.3 mg/kg, i.n., inhalation). For nasal instillation and oral administration to the mice, PIO solution was prepared by solubilizing the drug in DMSO followed by dilution with 0.3 M 2-hydroxypropyl-β-cyclodextrin. For inhalation, PIO NP was suspended in 0.22 μm-filtered water. For the treatment group (A), a dosing volume of 20–30 µL was intranasally administered to each mouse in standardized steps using thin gel loading pipette tips [25]. For example, if a dosing volume of 24 µL was required, we loaded the pipettor with 6 µL, administered to the left nostril first 2 small droplets with about half the volume (3 µL) per droplet, followed by a 15 s hold before repeating another 6 µL for the right nostril in the same manner. After allowing the mouse to rest in its cage for 2 min, the next 2 × 6 µL administration routine was repeated for both nostrils, thus giving a total dosing volume of 24 µL for one mouse. For group (B), the dose was administered via oral gavage. For group (C), mice were subjected to inhalation exposure using the system described earlier (Section 2.3.2). The aerosol was generated by nebulizing 7 mL resuspended PIO NP. Mice from each treatment group were sacrificed by CO_2_ euthanasia at pre-determined time points (0.5, 1, 1.5, 2, and 4 h) after dosing with 3–4 animals harvested per time point. The 4 h time point for mice treated by PIO NP was not presented as the PIO level in their brain samples was found to be below the lower limit of quantification for the HPLC method used to determine PIO level in brain samples. Blood samples were collected via cardiac puncture. Whole brains were carefully removed, washed twice with normal saline solution, and dabbed gently to remove the extraneous fluid. Blood samples were immediately cooled in an ice bath and then centrifuged at 4000 *g* for 10 min to collect the plasma. All brain and plasma samples were kept at −20 °C immediately after collection and stored at −80 °C until analysis. On the day of analysis, all samples were thawed and kept on ice. Brain tissue samples were homogenized with Milli-Q water in a ratio of 1 part tissue mass to 1 part water. Analytes were extracted using protein precipitation by adding 4 volumes of crashing solution (containing 3 µg/mL rosiglitazone as an internal standard in 1:1 mixture of methanol and acetonitrile). All samples were vortex-mixed at high speed for 5 min and centrifuged at 20,000 *g* for 30 min at 4 °C to pellet the precipitated protein. Supernatants were then transferred to glass vials for analysis using reversed-phase HPLC. Analyte/IS peak area ratios of spiked plasma, and brain samples were used to construct linear calibration curves for each sample matrix accordingly, which were then used to quantify PIO concentrations in experimental samples. Standard unpaired one-way ANOVA was applied for statistical comparison of in vivo experimental data and Tukey-adjusted *p*-value was used to determine significance. Significance was defined as *p* ≤0.05.

### 2.4. Cell Culture

Chinese hamster ovary (CHO) cells stably transfected with mouse Aβ precursor protein 695 (CHO-APP_695_) cells were used as the AD cell model for our in vitro experiments. Cells were incubated at 37 °C in the presence of 5% CO_2_ for all cell culture work. Cells were maintained in DMEM culture medium (Sigma D1152) containing 10% FBS and supplemented with 100 units/mL of penicillin and 100 μg/mL of streptomycin. They were standardized at the 3rd passage and harvested for plating when they reached log-phase growth. Cells were plated in DMEM low glucose culture medium (Sigma D2902) containing 10% FBS and the same supplements as the maintenance medium.

### 2.5. Analysis of In-Vitro Mitochondrial Respiration

CHO-APP_695_ cells were seeded in XF24 cell culture microplates (Agilent Seahorse) at a density of 10 × 10^3^ cells/well. To study treatment effects of PIO and PIO NP, additional groups of CHO-APP_695_ cells were seeded in culture media supplemented with PIO and PIO NP accordingly. Blank NP was dosed at a weight equivalent to that of PIO NP. Blank culture medium and 0.2% DMSO were also included as vehicle controls. After 24 h treatment, the culture medium in each well was removed and replaced with fresh culture medium, following which the cells were incubated for another 24 h. They were then washed twice, followed by the addition of unbuffered XF assay medium (Agilent Seahorse) containing 2 mM L-glutamine supplemented with 5.5 mM glucose and 1 mM sodium pyruvate. Cells were equilibrated in the assay medium by incubating for 30 min in a non-CO_2_ environment at 37 °C. Mitochondrial respiration was assessed using the Seahorse XFe24 Extracellular Flux Analyzer (Agilent Technologies, Santa Clara, CA, USA) by measuring the real-time oxygen consumption rate (OCR) of cells with a sequential injection of respiratory inhibitors from the XF Cell Mito Stress Test Kit (Agilent Seahorse) [26]. Mitochondrial OCR values were calculated as per the stress test kit manufacturer’s instructions and then normalized to total protein content, which was determined for each well using the Micro BCA Protein Assay Kit (Thermo Scientific Pierce, Rockford, IL, USA). For analysis of mitochondrial function in cells, each well was considered as a separate experiment. Statistical significance across multiple treatment groups was calculated by standard unpaired one-way ANOVA with *p*-values adjusted for multiple comparisons using the Tukey test. Significance was defined as *p* ≤0.05.

### 2.6. Effect of Long-Term Inhalation Exposure to PIO-Loaded PLGA-TPGS Nanoparticles

#### 2.6.1. Animal Husbandry and Long-Term Treatment

From Taconic (USA), 20 female Tg2576 mice were obtained at 3 months of age. Female mice were used in this long-term study because male Tg2576 mice are prone to marked aggression towards cagemates, and animals are preferably socially housed for behavioral assessments. The transgenic mice were housed under the same conditions as described in Section 2.3.1.

These 20 female 3-month-old (MO) Tg2576 mice were divided equally into 3 treatment groups and 1 non-treated negative control group. The 3 treatment groups were: (A) Oral vehicle (non-drug-containing peanut butter (PB) pellets), (B) oral PIO (drug-loaded PB pellets, 18 mg/kg), and (C) inhaled PIO-loaded PLGA-TPGS nanoparticles (estimated 0.3 mg/kg). The oral PIO dose was chosen based on previous studies [27]. For group (C), the aerosol generation system used for mouse inhalation was the same one as described earlier in Section 2.3.2. All dosing formulations were freshly prepared on a weekly basis. Mice in these 3 treatment groups were dosed for 4 months and then subjected to behavioral assessments. Meanwhile, the non-treated Tg2576 control group was subjected to behavioral testing at 3 months old. To minimize carryover effects from prior tests, each group of mice was subjected to the behavioral test battery only once.

#### 2.6.2. Preparation of PIO-Containing PB Pellets

PIO-containing PB pellets were prepared every week by calculating the average body weight of mice in the oral PIO treatment group and then determining the amount of drug per 10 *g* of PB required to formulate the concentration of drug that was equivalent to 18 mg/kg PIO in PB matrix. PB chips (Reese’s, Hershey, PA, USA) were heated to 85 °C in a clean glass mortar using a heating block. The required amount of drug was mixed with the melted PB chips using geometric dilution to create a homogenous mixture. The resulting warm, drug-laden PB mixture or warm PB melt without the drug (vehicle) was carefully dripped onto a small piece of aluminum foil placed on a weighing scale to make approximately 100 mg PB pellets. The pellets were rapidly frozen at −20 °C to consolidate their shape and stored in sealed polystyrene petri dishes at −80 °C until use.

#### 2.6.3. Acclimation to PB Pellets and Inhalation Exposure

Prior to the initiation of our long-term study, Tg2576 mice in the oral treatment groups (A) and (B) were introduced to the taste of blank, i.e., non-drug-containing PB pellets by using a small spatula to present a pellet to the mouse through the top of its cage. This was carried out once daily for 7 consecutive days. Overnight food deprivation was not necessary. By the end of the training week, all the mice successfully overcame their innate avoidance of novel stimuli, i.e., feeding the PB pellet. Average pellet consumption time was less than 1 min per mouse. In this study, no mouse failed to consume the PB pellet in longer than 2 min during the habituation phase. Voluntary feeding by mice in the oral groups was exemplified by the supplementary material Video S1. On the other hand, Tg2576 mice in the PIO NP treatment group (C) were also acclimated to restraint in the inhalation exposure tubes in about one week. Animals were trained to enter and remain in these tubes until released. During acclimation, mice were subjected to an aerosol generated by nebulizing 7 mL normal saline solution. Time spent by the animals in the inhalation exposure tubes was increased up to 30 min at a gradual rate over the training week.

#### 2.6.4. Spontaneous Activity in Open Field Test after 2-Month Treatment

Around halfway through the course of our 4-month treatment schedule, we noticed some behavioral differences among the 3 treatment groups (A), (B), and (C). Specifically, Tg2576 mice in the oral vehicle control group were observed to be generally more hyperactive than the ones in the oral drug and inhalation exposure groups. To quantify the observed differences, a basic open field test was performed to assess their spontaneous activity. Briefly, mice were allowed to habituate in a quiet environment for at least 30 min with free access to food and water. The animals were then transferred into a square open field cage (40 cm × 40 cm) and allowed to explore freely for at least 30 min. At a different facility, Tg2576 mice in the non-treated 3-month-old control group were tested in a 50 cm × 50 cm open field cage when they were subjected to the open field test at their arrival age. Locomotor activity was video-recorded and analyzed using the TopScan Behavior Analyzing System (Cleversys, Reston, VA, USA). The activity was compared in terms of total distance moved adjusted for arena size.

#### 2.6.5. Open Field Test at the End of the 4-Month Treatment

Upon conclusion of the entire 4-month treatment schedule, the open field test was repeated for Tg2576 mice in the 3 treatment groups (A), (B), and (C) with a 50 cm × 50 cm open field cage. Locomotor activity was video-recorded and analyzed using TopScan. Animal activity was compared in terms of total distance moved.

#### 2.6.6. Forced Alternation in Y-Maze

A symmetrical Y-maze with 3 arms fixed at 120° from each other was used but with 1 arm blocked. A single mouse was placed into the end of the start arm, facing the wall and away from the center. The animal was then allowed to explore the 2 open arms of the Y-maze for 10 min, while entry into the 3rd arm was blocked. After the training trial, the mouse was returned to its home cage for a 30 min inter-trial interval. During the probe trial, the blockage in the 3rd arm was removed, the mouse was again placed into the start arm and then allowed to access all 3 arms of the maze. All trials were video-recorded, and the total time that the animal spent in each arm was determined using TopScan. Animals with preserved cognitive function would tend to remember the previously blocked arm and preferred to spend more time in the novel arm during the probe trial.

#### 2.6.7. Spontaneous Alternation in Y-Maze

A single mouse was placed in the center of a symmetrical Y-shaped maze with 3 arms fixed at 120° from each other. The animal could freely explore all 3 arms. If the mouse had an intact spatial working memory, it will have a reduced tendency to enter the recently visited arm. TopScan was used to score the number of arm entries and to determine the number of alternations from the sequence of arm choices. A mouse was considered as having entered an arm when all 4 limbs were within the arm. Spontaneous alternation was expressed as a percentage and corresponded to the ratio of arm choices differing from the previous 2 choices divided by the number of total choices minus 2. A higher alternation ratio was indicative of sustained cognition as the animal must remember which arm was entered last to not re-enter it.

#### 2.6.8. Statistical Analyses for Behavioral Study

Behavioral data were first subjected to D’Agostino–Pearson normality test due to the limited sample size employed in our behavioral study [28]. Because the data were not consistent with a Gaussian distribution, the non-parametric Kruskal–Wallis test was applied for statistical comparison with *p*-values adjusted for multiple comparisons using the Dunn’s test. For spontaneous alternation in Y-maze, data were also analyzed using the Wilcoxon signed-rank test to compare each group’s percent alternation to a theoretical chance value of 50%. Significance was defined as *p* ≤0.05.

### 2.7. ^1^H-NMR-Based NMR Metabolic Profiling

#### 2.7.1. Sample Collection

Upon completion of their behavioral tests, all Tg2576 mice were sacrificed by CO_2_ euthanasia, and their blood samples were collected via cardiac puncture into Eppendorf tubes supplemented with 3 μL heparin. Blood samples were immediately cooled in an ice bath and centrifuged at 4000 *g* for 10 min to collect the plasma. Plasma samples were then aliquoted from the supernatants into clean Eppendorf tubes. Transcardial perfusion with normal saline was performed on the sacrificed mice to remove traces of blood from their organs before whole brain tissues were carefully removed from their skulls. Immediately after the whole brain was collected, different brain parts (cerebellum, cortex, hippocampus, and midbrain) were meticulously dissected and transferred into pre-labeled clean Eppendorf tubes. Samples were snap-frozen in liquid nitrogen immediately after collection and kept on dry ice. All biological samples were stored at −80 °C until further sample preparation.

#### 2.7.2. Extraction and Sample Preparation for NMR

Intact frozen brain tissue samples were kept on dry ice and weighed. 10 μL ice-cold Milli-Q water was added per mg frozen tissue sample and lysed using bead homogenizer (Next Advance Bullet Blender 24 Gold). 200 μL of each tissue homogenate was then transferred into new tubes for subsequent extraction. 200 μL plasma samples were used without dilution for extraction. Metabolites were extracted using protein precipitation by adding 4 volumes of ice-cold crashing solution (1:1 mixture of methanol and acetonitrile). All mixtures were vortex-mixed at high speed for 5 min, followed by centrifugation at 14,000 *g* for 20 min at 4 °C to pellet the precipitated protein. Supernatants were then transferred into glass vials and evaporated to dryness using a centrifugal evaporator (Genevac EZ-2 Elite), followed by overnight freeze-drying (Martin Christ Alpha 1-4 LDplus). Dried samples were stored at −80 °C until NMR acquisition.

Samples for ^1^H NMR spectroscopy were resuspended in 580 μL deuterated buffer (0.1 M sodium phosphate buffer at pH 7.4 containing 0.1 mM TSP-d_4_ and 0.7 mM sodium azide dissolved in deuterium oxide), vortexed and centrifuged at 12,000 *g* for 5 min at 20 °C. 550 μL supernatant was then transferred into a clean NMR tube (Norell S-5-800-7) for acquisition.

#### 2.7.3. Data Acquisition and Spectral Processing

NMR spectra were acquired at 298 K using a Bruker AVANCE 800 MHz system equipped with a 5 mm TXI cryoprobe. To remove the effect of macromolecules on spectral broadening and to suppress the resonance from water, a Carr–Purcell–Meiboom–Gill pulse sequence with presaturation was applied as a *T*_2_ filter. For each sample, 512 transients were collected into 64 K data points using a spectral width of 12,820 Hz, an acquisition time of 2.56 s, a relaxation delay of 3 s, and a pulse width of 11.30 μs. Using TopSpin 2.1 (Bruker Biospin, Coventry, UK), all NMR spectra were manually phased, baseline corrected, and shift referenced to TSP at 0 ppm. These pre-processed spectra were then imported into Chenomx NMR Suite 7.1 (Chenomx Inc., Edmonton, AB, Canada) for spectral binning. Binning was performed from 0.04 to 10 ppm with a bin size of 0.04 ppm but with the water (4.68–4.88 ppm) and methanol (3.32–3.36 ppm) regions excluded. Binning data were normalized based on the total binned area in each file.

#### 2.7.4. Multivariate Data Analysis of Metabolic Data Matrix

Normalized data were imported into SIMCA 15 (Umetrics; Sartorius Stedim, Umeå, Sweden) for multivariate data analysis with separate analyses carried out for plasma and the different brain parts (cerebellum, cortex, hippocampus, and midbrain). Data were first Pareto-scaled and mean-centered before employing principal component analysis (PCA) to obtain an overview of the observations and identify outliers in the data. If clustering trends were observed among the analyzed samples, the data were subsequently subjected to orthogonal projections to latent structures discriminant analysis (OPLS-DA) to build a discriminant model. Permutation testing was used to check for model validity and potential overfitting. Cross-validated ANOVA (CV-ANOVA) *p*-value was calculated as a measure of significance for the observed group separation in OPLS analysis. Looking at the *R*^2^ and *Q*^2^ values as well as whether *p*[CV-ANOVA] ≤0.05, we proceeded to generate from the constructed OPLS-DA model a list of potential discriminant metabolites for further analysis using variable influence on projection (VIP) score >1.0 and absolute p(corr) >0.5 as inclusion criteria [29]. These metabolites were first identified with reference to the Chenomx spectral reference libraries as well as published literature [30,31,32] and subsequently quantified by computer-assisted manual fitting in the Chenomx software. The 2-tailed independent *t*-test was used for statistical comparison of these potential discriminant metabolites between the reference group and pooled treatment groups, and a Bonferroni-adjusted *p*-value was used to determine significance. The fold-change (FC) value for each discriminant metabolite as a ratio of its mean level in the pooled treatment groups relative to that of the reference group was also calculated. Next, to find out which relevant metabolic pathways were altered between the 2 comparison groups, the identified discriminant metabolites were uploaded onto MetaboAnalyst 4.0, a web-based metabolomics tool suite [33] and analyzed using the ‘Pathway Analysis’ module. We selected the *Mus musculus* (KEGG) pathway library and specified ‘Global Ancova’ and ‘Relative-betweenness Centrality’ algorithms for pathway enrichment analysis and pathway topology analysis, respectively. Pathways with an impact value greater than 0.1 and a Holm–Bonferroni-adjusted *p*-value less than 0.05 were considered most relevant [34].

## 3. Results

### 3.1. PIO-Loaded PLGA-TPGS Nanoparticles have Spherical Shape and are Stable When Resuspended

**Figure 1:**
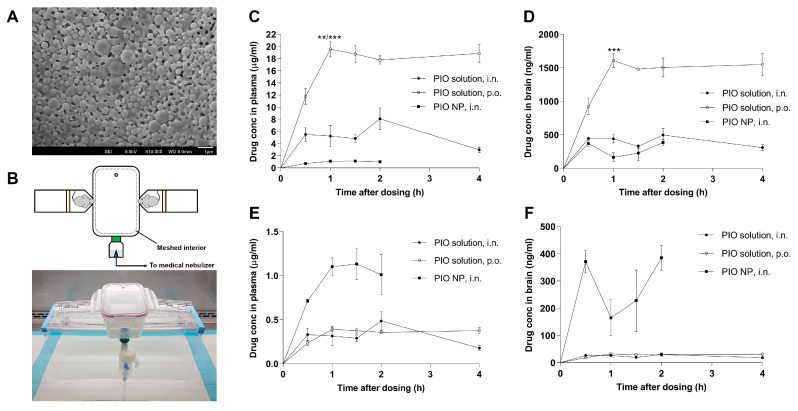
(**A**) Field emission scanning electron microscopy (FESEM) image of Pioglitazone (PIO)-loaded poly(lactic-co-glycolic acid) (PLGA) nanoparticles emulsified by D-α-Tocopherol polyethylene glycol succinate (TPGS). (**B**) Schematic diagram and photograph of the assembled inhalation system. (**C**) PIO concentrations versus time plots for plasma samples harvested from mice given PIO solution (●, 5 mg/kg, i.n., nasal instillation), PIO solution (○, 15 mg/kg, p.o.) and PIO NP (■, estimated 0.3 mg/kg, i.n., inhalation), respectively. (**D**) PIO concentrations versus time plots for brain samples. (**E**,**F**) Dose-normalized PIO concentrations versus time plots for plasma and brain samples, respectively, for comparison. Data are represented as mean ± SE with *N* = 3–4 C57BL/6 mice sacrificed per time point. ** indicates *p* < 0.01; *** indicates *p* < 0.001 when compared against the C_max_ points of the other treatment groups.

The particle size of the drug-loaded PLGA-TPGS nanoparticles remained relatively stable after reconstituting to 1 mg nanoparticles per mL in Milli-Q water and standing at room temperature (454.9 ± 31.9 nm, PDI = 0.432, pH = 5.4). The surface charge of the drug-loaded nanoparticles was characterized by measuring zeta potential (−26.8 ± 1.9 mV). Drug loading determined by HPLC was 10 µg drug per mg lyophilized powder. The morphology of the drug-loaded nanoparticles was examined using FESEM (Figure 1A), revealing solid, spherical particles with a smooth surface. While PLGA-based nanoparticles often offer high drug encapsulation efficiency, the drug loading was generally quite low (around 1%) [35], which was consistent with the level achieved in our formulation. The surface charge of our PIO-loaded PLGA nanoparticles was measured to be −26.8 mV, which is in agreement with the literature value [36]. Surface charge is important for maintaining the stability of nanoparticles in suspension as lower absolute values of zeta potential implies colloidal instability, which could lead to aggregation of nanoparticles.

### 3.2. Inhalation Exposure to PIO-Loaded PLGA-TPGS Nanoparticles Revealed Comparatively Lower Systemic Exposure than Simple Nasal Instillation or Oral Administration of the PIO Solution

Figure 1B shows the experimental setup that was used for exposing mice to an aerosol generated from the nebulization of our PIO NP formulation. The profiles of mean plasma and brain concentrations of PIO versus time are shown in Figure 1C,D, respectively. Relevant pharmacokinetic parameters are summarized in Table 1. Mice given oral PIO solution showed significantly higher plasma concentration maximum (C_max_, 19.55 ± 1.26 μg/mL) than did mice administered PIO solution by nasal instillation (8.09 ± 1.80 μg/mL, adjusted *p* = 0.0019) and PIO NP by nasal inhalation (1.13 ± 0.18 μg/mL, adjusted *p* = 0.0002). Significantly higher brain C_max_ was also observed for mice given oral PIO solution (1609 ± 104.9 μg/mL) compared to mice given PIO solution by nasal instillation (498 ± 96.5 μg/mL, adjusted *p* = 0.0002) and PIO NP by nasal inhalation (385 ± 45.5 μg/mL, adjusted *p* = 0.0001). The peak time (T_max_) of plasma drug concentration for mice administered oral PIO solution was 1 h. For mice in the i.n. PIO NP and i.n. PIO solution treatment groups, T_max_ was achieved slower at 1.5 h and 2 h, respectively. T_max_ of brain PIO concentration for mice from both intranasal treatment groups was 2 h after drug administration, whereas it peaked at 1 h for mice in the oral treatment group. The area-under-the-curve (AUC)_0–4 h_ values of plasma and brain PIO were 66.1 μg·h/mL and 5.44 μg·h/mL, respectively, for mice administered oral PIO solution. For mice that received PIO solution by nasal instillation, the plasma and brain PIO AUC_0–4 h_ values were 20.1 μg·h/mL and 1.52 μg·h/mL, respectively. For mice that were given PIO NP by inhalation, their plasma and brain PIO AUC_0–2 h_ values were 1.73 μg·h/mL and 0.48 μg·h/mL, respectively. Overall, the brain C_max_ after oral dosing of PIO solution was about four times higher than that after intranasal dosing of PIO NP. However, the dose difference between oral and inhalation dosing was around 50-fold, suggesting that the inhalation of PIO NP may be much more efficient in delivering the drug to the brain. This difference was exemplified by the dose-normalized profile of mean brain concentrations of PIO versus time shown in Figure 1F. This was also corroborated by the AUC_brain_-to-AUC_plasma_ ratio, which was another indication of the extent of brain uptake [37]. Among the three treatment groups, mice that received PIO NP by inhalation showed the highest AUC_brain_-to-AUC_plasma_ ratio (27.8%) compared to mice that were given PIO solution by nasal instillation (7.58%) and oral PIO solution (8.23%).

### 3.3. CHO-APP_695_ Cells Treated with PIO-Loaded PLGA-TPGS Nanoparticles Show Augmented Mitochondrial Spare Respiratory Capacity

**Figure 2:**
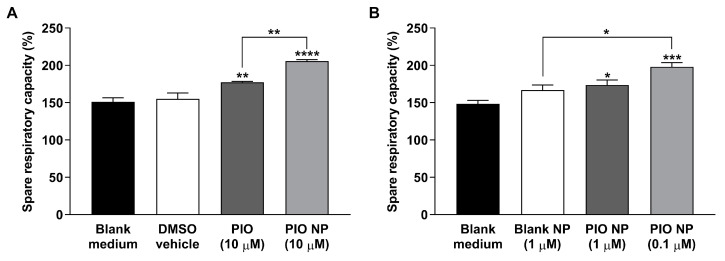
(**A**) Pre-treatment with 10 μM PIO and 10 μM PIO-loaded PLGA-TPGS nanoparticles increases mitochondrial spare respiratory capacity in CHO-APP_695_ cells, conferring the cells greater ability to resist stress. (**B**) Lower doses of PIO NP (1 μM and 0.1 μM) pre-treated CHO-APP_695_ cells exhibit significantly increased spare respiratory capacity as well. OCR data are normalized to total protein content and presented as mean ± SE (*N* = 5). Outliers have been omitted. * indicates *p* < 0.05; ** indicates *p* < 0.01; *** indicates *p* < 0.001; **** indicates *p* < 0.0001 when compared against the blank medium control unless otherwise indicated.

Next, we investigated whether 10 μM of our nanoparticulate drug formulation had any effect on mitochondrial respiratory activity in CHO-APP_695_ cells using extracellular flux analysis. The same concentration of PIO has been shown to elicit therapeutic responses in in vitro experiments [38]. The vehicle 0.2% DMSO used for the PIO solution was confirmed not to affect mitochondrial respiration significantly, hence, all treatment groups were compared to the blank culture medium control for uniformity. After discounting non-mitochondrial respiration, CHO-APP_695_ cells pre-treated with 10 μM PIO and 10 μM PIO NP were both found to exhibit significantly increased reserve respiratory capacity compared to the blank control (Figure 2A). Pre-treatment with 10 µM PIO solution showed a significantly higher spare respiratory capacity of 177.24 ± 1.19 percent as compared to the control’s spare respiratory capacity of 151.10 ± 5.38 percent (adjusted *p* = 0.0083). Significantly higher reserve respiratory capacity was also observed for cells pre-treated with 10 µM PIO NP (205.82 ± 1.70 percent, adjusted *p* < 0.0001). Specifically, pre-treatment with 10 µM PIO NP was found to significantly increase the reserve respiratory capacity of CHO-APP_695_ cells even more than cells pre-treated with 10 µM PIO solution (adjusted *p* = 0.0041).

Using information gained from our preliminary investigation of drug distribution to the brain (Section 3.2), we also investigated whether the in vivo PIO concentration (1 μM) achieved in the brain afforded by intranasal administration of our nanoparticulate drug formulation had any effect on mitochondrial respiratory activity in the same AD cell model. To ascertain that treatment effects observed were not due to the nanoparticulate carrier itself, an additional treatment group of CHO-APP_695_ cells pre-treated with 1 μM blank NP was included, although no significant differences in reserve respiratory capacity were observed for cells pre-treated with blank NP relative to the control group (Figure 2B). On the other hand, CHO-APP_695_ cells pre-treated with drug-loaded nanoparticles were found to exhibit significantly increased reserve respiratory capacity compared to the blank medium control. Pre-treatment with 1 µM PIO NP showed a significantly higher spare respiratory capacity of 173.69 ± 6.71 percent as compared to the control group’s spare respiratory capacity of 148.48 ± 4.60 percent (adjusted *p* = 0.0416). Significantly higher reserve respiratory capacity was also observed for cells pre-treated with a lower dose of 0.1 µM PIO NP (197.95 ± 5.69 percent, adjusted *p* = 0.0001). Intriguingly, 0.1 µM PIO NP showed a significant treatment difference when compared to 1 μM blank NP, which had a reserve respiratory capacity ratio of 166.75 ± 6.84 percent (adjusted *p* = 0.0103). However, there was no significant treatment difference seen between the 1 µM PIO NP and 1 µM blank NP groups. Neither was significant treatment difference observed between the 1 µM PIO NP and 0.1 µM PIO NP groups, suggesting that the gain in reserve respiratory capacity offered by the lower dose may be marginal.

### 3.4. Long-Term Oral PIO and PIO NP Inhalation Treatments Attenuate Hyperactivity in AD Mice

**Figure 3:**
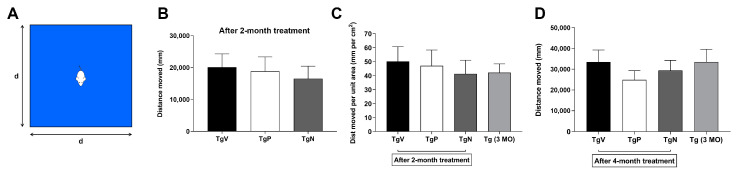
(**A**) Schematic diagram of a basic open field test setup. (**B**) Open field test results after 2-month treatment shown as total distance traveled by oral vehicle-treated Tg2576 mice (TgV, 5-month-old), oral PIO-treated Tg2576 mice (TgP, 5-month-old) and PIO NP inhalation-treated Tg2576 mice (TgN, 5-month-old) in a 40 cm × 40 cm open field cage. (**C**) Total distance traveled adjusted for arena size to compare with non-treated Tg2576 mice (Tg, 3-month-old) that were earlier trialed in a 50 cm × 50 cm testing arena. (**D**) Open field test results after completion of 4-month treatment shown as total distance traveled in a 50 cm × 50 cm open field cage. Data are represented as mean ± SE.

Around 2 months into the treatment period, Tg2576 mice that received oral vehicle treatment were observed to be generally more hyperactive than the ones that received oral PIO and PIO NP via inhalation. A basic open field test (diagram of the setup shown in Figure 3A) was carried out to assess their spontaneous activity to quantify the observed differences. Figure 3B shows the total distance traveled by Tg2576 mice in a 40 cm × 40 cm open field cage. Oral PIO and PIO NP inhalation-treated animals traveled overall shorter distances (18,825 ± 4513 mm, 16,515 ± 3922 mm, respectively) than oral vehicle-treated ones (20,086 ± 4193 mm). After adjusting for arena size (Figure 3C), Tg2576 mice that were given i.n. PIO NP and oral PIO treatment showed relatively lower spontaneous activity levels (41.3 ± 9.8 and 47.1 ± 11.3 mm per cm^2^, respectively) than the oral vehicle-treated mice (50.2 ± 10.5 mm per cm^2^). Tg2576 mice given i.n. PIO NP treatment showed a slightly lower spontaneous activity level than their younger non-treated counterparts, whose activity level was found to be 42.1 ± 6.3 mm per cm^2^. Conversely, oral PIO-treated Tg2576 mice exhibited higher spontaneous activity levels than the 3-month-old Tg2576 mice. Although the group differences were not statistically significant, the general trend observed in this open field test suggests that these drug treatments may help AD mice to reduce hyperactivity and maintain their ability to habituate to new environments. Upon conclusion of the entire 4-month treatment schedule, Tg2576 mice that received oral PIO and i.n. PIO NP were again observed to exhibit reduced spontaneous activity levels (24,885 ± 4381 and 29,407 ± 4855 mm, respectively) when compared to the oral vehicle-treated Tg2576 mice (33,493 ± 5792 mm) as shown in Figure 3D. Compared to the younger non-treated mice (33,497 ± 6159 mm), AD mice in both drug-treated groups demonstrated reduced spontaneous activity levels. Similarly, this general trend suggests that oral PIO and i.n. PIO NP treatments may help AD mice to maintain their ability to habituate to new environments, although the mice that were given oral PIO now exhibited lower spontaneous activity than those that received i.n. PIO NP as compared to when the animals were previously assessed 2 months into the treatment period.

### 3.5. Long-Term Oral PIO and PIO NP Inhalation Treatments have No Obvious Effect on Spontaneous Alternation Behavior in AD Mice

**Figure 4:**
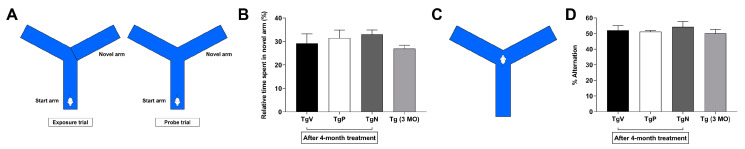
(**A**) Schematic representation of setup for the forced alternation in the Y-maze. (**B**) Time spent in the novel arm during the probe trial. Tg2576 mice (7-month-old) in the TgP and TgN groups had completed their 4-month treatment schedule with age-matched oral vehicle-treated mice in the TgV group. (**C**) Schematic representation of setup for the spontaneous alternation in the Y-maze. (**D**) Percent alternation in the three free arms. Data are represented as mean ± SE.

Setup for forced alternation in Y-maze is depicted in Figure 4A. Figure 4B shows the relative time spent in the novel arm during the probe trial. After 4 months treatment with oral PIO and i.n. PIO NP, Tg2576 mice were observed to spend more time in the novel arm (31.5 ± 3.4 and 33.0 ± 1.9 percent, respectively) than those that received oral vehicle treatment (29.2 ± 4.0 percent), although the differences were not found to be statistically significant. Their younger non-treated counterparts generally spent the least amount of time in the novel arm (27.0 ± 1.4 percent). Figure 4C illustrates the setup for the Y-maze spontaneous alternation test. Tg2576 mice that received i.n. PIO NP treatment showed 54.2 ± 3.5 percent alternation (Figure 4D). Oral PIO and oral vehicle-treated mice showed 51.2 ± 0.8 and 52.1 ± 3.0 percent alternation, respectively. The non-treated 3-month-old mice exhibited the least tendency to alternate the free arms on successive choice (50.0 ± 2.6 percent alternation).

### 3.6. Metabolic Profiling Revealed Differences in Amino Acid Metabolism in Cortex Region after Long-Term Oral PIO and PIO NP Inhalation Treatments

As metabolic perturbations are known to be region-specific in the brains of various AD mouse models [32,39], different parts of the brain (cerebellum, cortex, hippocampus, and midbrain) were collected separately for metabolic profiling. We also analyzed the plasma metabolome of our Tg2576 mice as there was no plasma-specific metabolomics analysis reported for this AD mouse model. The oral vehicle-treated Tg2576 mice were employed as the reference group as age is known to be a contributing factor to metabolome variation. Because no differences were observed when the metabolic datasets for oral PIO and PIO NP inhalation groups were compared against each other using PCA, we pooled these two groups together as the PIO-treated group for greater power when comparing against the reference group in multivariate data analysis.

**Figure 5:**
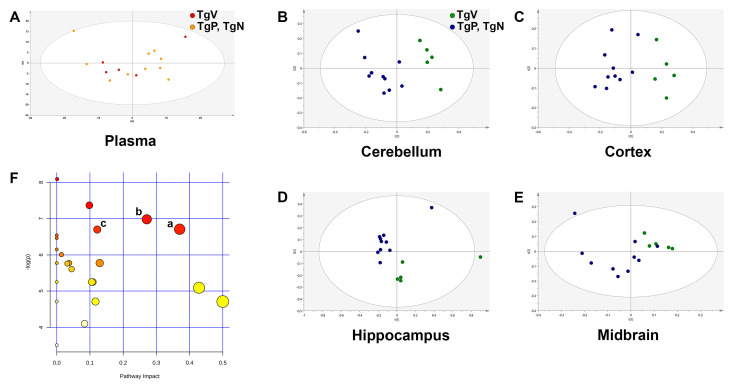
Principal component analysis (PCA) scores plots of metabolic profiles for the reference group (TgV, oral vehicle-treated Tg2576 mice) and pooled drug treatment groups (TgP, oral PIO-treated Tg2576 mice; TgN, PIO NP inhalation-treated Tg2576 mice) for (**A**) plasma, (**B**) cerebellum, (**C**) cortex, (**D**) hippocampus, and (**E**) midbrain matrix, respectively. (**F**) Pathway analysis overview with each node representing an altered metabolic pathway in the cortex of the pooled drug treatment groups versus the reference group. The three most relevant pathways are: (a) Alanine, aspartate, and glutamate metabolism; (b) glycine, serine, and threonine metabolism; (c) arginine and proline metabolism. The more upstream the metabolite is in the pathway, the higher impact value it will derive (*x*-axis), whereas the more number of ‘hits,’ i.e., metabolites affected in that biological pathway, the more important the pathway seems to be (*y*-axis).

Comparing age-matched female Tg2576 mice, PCA of plasma samples from the oral vehicle group and the drug-treated groups did not display any visible clustering trend, which indicates there is only a slight or no difference between their plasma metabolites (Figure 5A). Because no clear group separation was observed here, the OPLS-DA model was not constructed for the plasma matrix. This suggests that plasma in Tg2576 mice at around 7 months old could not yield much useful information regarding the underlying metabolic processes in this AD mouse model. On the other hand, PCA for cerebellum, cortex, and hippocampus matrices (Figure 5B–D) displayed relatively clear separation between the reference and treatment groups. However, this separation was not as clear for the midbrain matrix (Figure 5E). Nevertheless, OPLS-DA was carried out for all four brain regions to further enhance separation trends and thus identify potential discriminant metabolites that contributed to the difference. Table 2 shows the number of components employed in the OPLS-DA model construction as well as the *R*^2^(cum), *Q*^2^(cum), and *p*[CV-ANOVA] values for the respective brain regions. The number of components employed in model construction was related to the degree of overfitting. The *R*^2^ value indicates how well the model explains the dataset, whereas the *Q*^2^ value provides a measure of the predictive power of the model. OPLS-DA model for cerebellum metabolic data was constructed with 1 predictive + 1 orthogonal component (*R*^2^(cum) = 0.945, *Q*^2^(cum) = 0.887, *p*[CV-ANOVA] = 0.00010). OPLS-DA for cortex metabolic data led to a model with one principal component and gave *R*^2^(cum) and *Q*^2^(cum) values of 0.861 and 0.806, respectively (*p*[CV-ANOVA] = 0.00005). OPLS-DA model for the hippocampus dataset was built with 1 predictive + 1 orthogonal component (*R*^2^(cum) = 0.894, *Q*^2^(cum) = 0.818, *p*[CV-ANOVA] = 0.00101). The high *Q*^2^(cum) values obtained for these three brain regions indicated that the separation between metabolic profiles of the reference and treatment groups was robust. The *p*[CV-ANOVA] values calculated for these three brain regions were all smaller than 0.05, indicating that the group separation in OPLS-DA analyses was significant. Meanwhile, the OPLS-DA model for midbrain metabolic data was constructed with comparatively more components (1 predictive + 4 orthogonal). Although the model gave relatively high *R*^2^(cum) and *Q*^2^(cum) values (0.997 and 0.842, respectively), *p*[CV-ANOVA] was calculated to be 0.05442, suggesting that the group separation for the midbrain matrix may not be significant.

Based on these parameters of the OPLS-DA models constructed, a list of potential discriminant metabolites satisfying VIP >1.0 and |p(corr)| >0.5 that contributed to the separation was identified for the cerebellum, cortex, and hippocampus region, respectively (Table 3). Because the *p*[CV-ANOVA] value exceeds 0.05, the OPLS-DA model built using the midbrain dataset was not used for the discovery of discriminant metabolites. For cerebellum samples, a list of 8 potential discriminant metabolites that contributed to separation was identified. Of these eight, only γ-aminobutyrate (GABA) was elevated in the pooled treatment groups, whereas the remaining seven were lower than the reference group, although none achieved statistical significance when means were compared between the oral vehicle-treated and drug-treated transgenic mice. For hippocampus samples, only 1 metabolite (ascorbate) out of the 10 putatively identified metabolites (4 upregulated and 6 downregulated) was found to be significantly changed in the pooled drug treatment groups versus the reference group. Most of the significant metabolic alterations were found in the cortical metabolic profiles (11 out of 15 putatively identified metabolites), suggesting that this was the most affected region in Tg2576 mice treated with oral PIO and i.n. PIO NP compared to oral vehicle-treated controls. Significantly elevated metabolites in the cortex of drug-treated mice are valine, alanine, N-acetylaspartate (NAA), GABA, creatine, choline, taurine, myo-inositol (mIns), phosphoethanolamine (PE), ascorbate, and adenosine. Pathway investigation of the potential discriminant metabolites identified in the three different brain regions also revealed that the significantly impacted pathways in the drug-treated groups versus oral vehicle group were present in the cortex. The three most relevant pathways are: (a) Alanine, aspartate, and glutamate metabolism (impact value = 0.37, adjusted *p* = 0.0293), (b) glycine, serine and threonine metabolism (impact value = 0.27, adjusted *p* = 0.0232) and (c) arginine and proline metabolism (impact value = 0.12, adjusted *p* = 0.0293) (Figure 5F).

## 4. Discussion

PIO is a poorly soluble drug, which renders a simple drug solution unsuitable for long-term administration for treatment of a chronic condition such as AD. As such, it was essential for us to devise a safe and stable drug delivery solution to facilitate long-term intranasal administration of PIO. PLGA has emerged as one of the most promising biomaterial for nanostructure-based drug delivery systems [35,40]. It has a long history of use in biomedical applications owing to its excellent biocompatibility and biodegradability [41]. PLGA has also been approved by the FDA as matrix for drug delivery [42], further highlighting its value and translatability in biomedical applications. We observed no significant histopathological changes in mice chronically exposed by inhalation to our PLGA-based nanoparticulate formulation (data not shown), suggesting that these nanoparticles are safe for long-term use. The relatively low drug loading is probably the major hurdle limiting the use of drug-loaded PLGA-based nanoparticles, however, our metabolic profiling results demonstrates that low-dose PIO nanoparticles delivered via the intranasal route can also affect energy metabolism in the brain comparable to oral PIO administered at a much higher dose.

From our preliminary investigation of drug distribution to the brain, the brain C_max_ after oral dosing of PIO solution was found to be about four times higher than that after intranasal dosing of PIO NP. However, the dose difference between oral and inhalation dosing was around 50-fold. Furthermore, among the three treatment groups, mice that received PIO NP by inhalation showed at least triple the AUC_brain_-to-AUC_plasma_ ratio compared to mice that were given PIO solution by nasal instillation and the oral route. Taken together, this indicates that inhalation of PIO NP may be much more efficient in delivering the drug to the brain. This also suggests that inhalation exposure to PIO NP may lower systemic exposure compared to simple nasal instillation or oral administration of the PIO solution, which is useful for harnessing the therapeutic effects of PIO in the brain while offering the advantage of lessening peripheral side effects associated with the drug given the controversy that PIO is associated with an increased risk of bladder cancer.

CHO cells have been used to screen potential therapeutic agents for treating neurodegenerative diseases [43]. Notably, transfected CHO cells overexpressing APP have been used as a cellular model to study the bioenergetic effects induced by endogenously produced Aβ peptides [44]. CHO-APP_695_ cells pre-treated with 10 μM PIO and 10 μM PIO NP were observed to exhibit significantly higher reserve respiratory capacity ratios, which is the difference between maximal and basal OCRs and serves as an indicator of how well the mitochondria within cells are able to increase ATP production in response to increases in energy demand [45]. Importantly, deficits in mitochondrial reserve respiratory capacity have been found to correlate to neuropsychological changes seen in patients with early stages of sporadic AD [46]. In this study, we noticed that pre-treatment with 10 µM PIO NP elevated the reserve respiratory capacity even more than 10 µM PIO solution, which could be due to the increased cellular uptake of TPGS-coated PLGA nanoparticles [47]. Previously, treatment of human neuron-like cells with 10 μM PIO was found to increase cellular respiration rates by inducing mitochondrial biogenesis [48]. It is well-established that PIO activates the peroxisome proliferator-activated receptor gamma (PPARγ) and upregulates the expression of the PPARγ coactivator 1α (PGC-1α), which is the master regulator of mitochondrial biogenesis [49,50]. Another mechanism that has been proposed for PIO’s ability to increase mitochondrial bioenergetics is its interaction with a novel mitochondrial membrane protein called mitoNEET [51], which has been identified to play a role in mitophagy [52]. There are recent studies suggesting that impaired mitophagy induces the accumulation of dysfunctional mitochondria, which in turn disrupts cellular bioenergetics and promotes AD pathology [53,54]. Typically, 10 μM PIO has been used to elicit therapeutic responses in in vitro experiments [38,48]. However, this concentration exceeds clinically achieved plasma levels [55] and is not likely to be attained in the brain considering the limited penetration of PIO across the BBB.

Interestingly, significantly higher reserve respiratory capacity ratios were observed even when the cells were pre-treated with lower doses of PIO NP during our attempt to find out whether the in vivo PIO concentration (1 μM) achieved in the brain afforded by intranasal administration of our nanoparticulate drug formulation had any effect on mitochondrial respiration. Previously, repeated dosing of 100 nM PIO in human neuroblastoma cells has been found to upregulate PGC-1α expression and promote mitochondrial biogenesis [56], and PPARγ stimulation using 100 nM to 1 μM PIO was also reported to promote neurite outgrowth [57], although mitochondrial integrity was found to be compromised at more than 20 μM [48]. Low doses of PIO have been found to improve cerebral functional connectivity in rodent brains, and the effect was least pronounced in the group treated with the highest PIO dose [58]. It was also reported that PIO 15 to 30 mg [59] but not 45 mg [14] has better pro-cognitive effects than placebo in AD patients. These suggest that the effect of PIO may have an optimal range, rather than a simple linear dose-dependent relationship.

We adopted the transgenic Tg2576 murine model for our long-term efficacy study. Tg2576 is an extensively characterized mouse model for AD that overexpresses the 695-amino acid isoform of human APP carrying the Swedish double mutation [60]. The cognitive function of Tg2576 mice was reported to decline in an age-dependent manner: 3-month-old Tg2576 are cognitively normal, 5-month-old Tg2576 are mildly cognitively-impaired, and 9-month-old Tg2576 are severely cognitively-impaired, and cognitive function continues to deteriorate as the animals age [61]. These mice demonstrate rapid increases in Aβ levels starting at 6 months old and marked deposition of Aβ in amyloid plaques between 9 and 12 months [62]. Reduced respiratory capacities were detected in brain mitochondria isolated from young Tg2576 mice [63,64], suggesting that impaired mitochondrial function occurs early in the disease for this AD mouse model. In this study, we chose to begin treatment in 3-month-old Tg2576 mice to investigate the effectiveness of starting treatment at an early stage of the disease before the overt accumulation of extracellular Aβ and onset of cognitive deficits in the animals. Moreover, it has been proposed that APP transgenic mouse models better model the asymptomatic phase of AD, and interventional studies using these mice should be considered in the context of disease prevention [65].

Interestingly, we noticed behavioral differences among the treatment groups halfway through the course of our 4-month treatment schedule. Specifically, Tg2576 mice in the oral vehicle group were observed to be generally more hyperactive than those in the oral drug and inhalation exposure groups. Therefore, we decided to carry out a basic open field test to quantify the observed differences. Compared to age-matched oral vehicle-treated Tg2576 mice, Tg2576 mice that received oral PIO and PIO NP via inhalation showed a general trend towards reduced spontaneous activity in the open field test 2 months into the treatment schedule as well as upon completion of treatment. This raises the possibility that PIO treatment may influence locomotor activity in this transgenic mouse model as Tg2576 mice have been reported to be more active in the open field task than transgene-negative controls [66]. Aberrant motor behavior was also reported to be correlated with cognitive impairment in AD patients [67]. However, we were not able to evaluate whether this difference was statistically different due to the high individual variations in our small sample size. It was reported that the sample size required to achieve statistically significant differences given the variability of disease outcomes in most AD mouse models is typically around 20 to 30 animals per group [68], and even larger numbers of animals are necessary to achieve higher statistical power [69]. Next, the relatively simple spontaneous alternation behavior test was used to assess memory retention. Tg2576 mice have been reported to be impaired in their tendency to spontaneously alternate arm-entry in a Y-maze [60,66]. Neither long-term treatment with oral PIO nor i.n. PIO NP was observed to have any visible effect on alternation rates compared to age-matched oral vehicle-treated controls. Our metabolic profiling results indicate that the cortex is the most significantly affected brain region in PIO-treated Tg2576 mice, however, rodent exploratory behavior measured by spontaneous alternation is also dependent on the integrity of multiple brain regions other than the cortex [70]. It is also possible that cognitive measures may be limited to surrogate outcomes as they do not faithfully reflect underlying disease processes.

As assessing locomotor activity and spontaneous alternation behavior turned out to be inadequate for distinguishing the efficacy of intranasal low-dose PIO NP in Tg2576 mice, to evaluate better the potential of this treatment strategy on metabolic dysfunction in AD, we further examined the metabolic profiles of extracts from the mouse plasma and brain tissues. The specificity of PIO’s effects on glucose metabolism in the cortex has been reported in different AD mouse models [10,12]. However, the reason as to why its effect is pronounced in this particular brain region remains unclear, although it is known that the cerebral cortex and hippocampus are the most affected brain regions in human AD [1]. For Tg2576 mice, metabolic changes were reported to appear first in the hippocampus and rhinal cortex as early as 1 month of age, whereas the cerebellum and midbrain were affected later at 11 months [32]. This is in line with our findings in which marked metabolic alterations were mainly observed in the cortex of PIO-treated Tg2576 mice, and in our case, the three most relevant pathways were all found to be associated with amino acid metabolism [71]. Notably, the dominance of these three amino acid pathways were reported in pathway enrichment analyses of AD, Parkinson’s disease, and amyotrophic lateral sclerosis [72]. The key metabolites that were involved in the altered pathways are alanine, NAA, GABA, creatine, and choline. The levels of these five metabolites were significantly elevated in the drug-treated groups compared to the reference group. Metabolic perturbations are known to be strongly influenced by factors such as the type of animal model used, brain region, and age, which complicates the comparison of data from different studies. Therefore, we attempted to compare our results to studies that also employed the Tg2576 mouse model. In old Tg2576 mice, in vitro and in vivo magnetic resonance spectroscopy showed that NAA was markedly reduced in the cortex compared to aged-matched wild-type littermates [73]. Alanine, GABA, and choline levels were also found to be decreased in these mice, although the differences were not statistically significant. Clinically, the NAA peak is often the most prominent signal detectable with proton magnetic resonance spectroscopy in human brains, and this metabolite was frequently found to be significantly lowered in various brain regions of AD patients, including the cortex [74,75]. Since NAA is an amino acid exclusively produced in neuronal mitochondria, it is widely regarded as a surrogate marker of neuronal health in neurologic disorders [74]. Of note, NAA levels were increased in our PIO-treated mice, highlighting the effect of PIO on mitochondrial metabolism in neurons. In another NMR-based metabolomic study performed on Tg2576 mice, alanine levels were found to be significantly reduced in the rhinal and frontal cortices of 11-month-old transgenic mice compared to their age-matched non-transgenic counterparts [32]. It was reported that alanine was utilized as one of the top alternative sources of energy to overcome inadequate glucose supply and energy crisis in neurodegenerative diseases such as AD [76]. In this case, alanine levels were increased after PIO treatment, suggesting that the brain cells may not be requiring this amino acid as compensatory fuel to sustain growth. Future metabolic profiling studies using alternative platforms such as gas chromatography-mass spectrometry can help to complement the findings of this study and contribute to the elucidation of metabolic processes affected in this AD mouse model. This is because the metabolic profiles of brain regions such as the cerebellum and hippocampus displayed clear signs of separation in their PCA scores plots, however, we were not able to pinpoint the metabolites that contributed significantly to the observed separation, likely because of the lower sensitivity of NMR. In this aspect, the higher sensitivity, resolution, and dynamic range of a mass spectrometry-based platform [77] could potentially help to identify these metabolites, thus improving the coverage of the metabolome.

## 5. Conclusions

To the best of our knowledge, this is the first behavioral phenotyping and metabolic profiling study of long-term inhalation exposure to a nanoparticulate formulation for PIO in a mouse model of AD pathology. In this study, we described the preparation of PIO-loaded PLGA nanoparticles for intranasal delivery to the brain. We also described the design of a simple and inexpensive aerosol generation system for nebulization of our nanoparticulate drug formulation for mouse inhalation. Inhalation exposure to PIO NP revealed comparatively lower systemic exposure than simple nasal instillation or oral administration of PIO solution, although the absolute amount of drug achieved in the brain was lower. However, findings from our behavioral and metabolic profiling experiments after long-term treatment indicate that drug efficacy may not be linearly proportional to the drug concentration in brain, i.e., even though the drug level achieved in the brain is 10-fold higher after oral administration, the drug effect is not 10 times better. This was corroborated by the significantly elevated reserve respiratory capacity observed even when AD cells were treated with lower doses of PIO NP. Although cognitive measures turned out to be inadequate for evaluating treatment efficacy in these mice, metabolic profiling of different brain regions revealed the specificity of PIO’s treatment effects on perturbed amino acid metabolism in the cortex of Tg2576 mice with marked increases in alanine and N-acetylaspartate levels, which supports the notion that PIO treatment exerts beneficial effects on impaired energy metabolism associated with AD. Although the number of animals used was relatively modest in this pilot investigation, and a larger sample size would confer greater statistical power for the behavioral phenotyping experiments, our results nonetheless suggest the potential of PIO NP inhalation to administer this drug intranasally at a much lower dose, which achieves a sufficient level in the brain to elicit metabolic benefits at an early stage of AD but with reduced systemic exposure given the persistent concern that chronic high-dose exposure to PIO has been associated with an increased risk of bladder cancer.

## Figures and Tables

**Table 1:** Pharmacokinetic parameters in plasma and brain of mice in the three treatment groups: (A) PIO solution (5 mg/kg, i.n., nasal instillation), (B) PIO solution (15 mg/kg, p.o.), and (C) PIO NP (estimated 0.3 mg/kg, i.n., inhalation).

Treatment Group	Plasma			Brain			$\frac{{\mathrm{AUC}}_{\mathrm{brain}}}{{\mathrm{AUC}}_{\mathrm{plasma}}}$
	C_max_ (µg/mL)	T_max_ (h)	AUC_0 → 4 h_ (μg·h/mL) ^a^	C_max_ (ng/mL)	T_max_ (h)	AUC_0 → 4 h_ (μg·h/mL) ^a^	
PIO, i.n.	8.09 ± 1.80 0.48 ± 0.11 ^b^	2	20.06 1.21 ^b^	498 ± 96.5 30 ± 5.8 ^b^	2	1.52 0.09 ^b^	7.58%
PIO, p.o.	19.55 ± 1.26 0.39 ± 0.03 ^b^	1	66.11 1.32 ^b^	1609 ± 104.9 32 ± 2.1 ^b^	1	5.44 0.11 ^b^	8.23%
PIO NP, i.n.	1.13 ± 0.18	1.5	1.73	385 ± 45.5	2	0.48	27.75%

^a^ AUC_0 → 2 h_ was calculated for treatment group (C). ^b^ Normalized to the lowest dose, i.e., 0.3 mg/kg.

**Table 2:** The orthogonal projections to latent structures discriminant analysis (OPLS-DA) model information for the cerebellum, cortex, hippocampus, and midbrain samples.

Brain Region	No. of Components	*R*^2^(cum)	*Q*^2^(cum)	*p*[CV-ANOVA]
Cerebellum	1 predictive + 1 orthogonal	0.945	0.887	0.00010
Cortex	1 predictive + 0 orthogonal	0.861	0.806	0.00005
Hippocampus	1 predictive + 1 orthogonal	0.894	0.818	0.00101
Midbrain	1 predictive + 4 orthogonal	0.997	0.842	0.05442

**Table 3:** List of potential discriminant metabolites that differentiate tissue samples of age-matched Tg2576 mice in the pooled drug treatment groups (TgP and TgN) from the reference group (TgV) for cerebellum, cortex, and hippocampus matrices.

Brain Region	Metabolite	*δ* (ppm)	Signal ^a^	FC	Adjusted *p* Value ^b^
Cerebellum	Lactate	1.31	*d,* CH_3_	0.96	NS
	NAA	2.01	*s,* CH_3_ (acetyl moiety)	0.95	NS
	Glutamate	2.12	*m,* CH_2_	0.98	NS
	GABA	2.28	*t,* CH_2_	1.09	NS
	Creatine	3.03	*s,* N(CH_3_)	0.98	NS
	GPC	3.21	*s,* N(CH_3_)_3_ (choline moiety)	0.82	NS
	Taurine	3.25	*t,* CH_2_	0.98	NS
	mIns	3.61	*t,* CH	0.96	NS
Cortex	Valine	1.03	*d,* CH_3_	1.76	0.000791
	Alanine	1.47	*d,* CH_3_	1.54	0.001229
	NAA	2.01	*s,* CH_3_ (acetyl moiety)	1.46	0.004118
	Glutamate	2.12	*m,* CH_2_	1.32	NS
	GABA	2.28	*t,* CH_2_	1.67	0.00077
	Creatine	3.03	*s,* N(CH_3_)	1.42	0.004823
	Choline	3.18	*s,* N(CH_3_)_3_	1.72	0.010769
	GPC	3.21	*s,* N(CH_3_)_3_ (choline moiety)	1.28	NS
	Taurine	3.25	*t,* CH_2_	1.42	0.032589
	Glycine	3.55	*s,* CH_2_	1.32	NS
	mIns	3.61	*t,* CH	1.39	0.007666
	PE	3.82	*m,* CH_2_	1.27	0.004332
	Lactate	4.09	*q,* CH	1.26	NS
	Ascorbate	4.49	*d,* CH	1.60	0.001417
	Adenosine	8.23	*s,* CH	2.00	<0.000001
Hippocampus	NAA	2.01	*s,* CH_3_ (acetyl moiety)	1.02	NS
	Glutamate	2.12	*m,* CH_2_	1.03	NS
	GABA	2.28	*t,* CH_2_	0.92	NS
	Aspartate	2.80	*dd,* CH_2_	0.93	NS
	Creatine	3.03	*s,* N(CH_3_)	0.94	NS
	GPC	3.21	*s,* N(CH_3_)_3_ (choline moiety)	0.86	NS
	Taurine	3.42	*t,* CH_2_	0.99	NS
	mIns	3.52	*dd,* CH	0.95	NS
	Lactate	4.09	*q,* CH	1.03	NS
	Ascorbate	4.49	*d,* CH	1.64	0.002485

^a^ Chemical shifts are reported with reference to TSP-d_4_ trimethyl singlet resonance at 0.00 ppm, and multiplicity definitions are: *s,* singlet; *d,* doublet; *t,* triplet; *q,* quartet; *m,* multiplet; *dd,* doublet of doublet. ^b^ *p*-values were calculated for mean comparison between the reference and pooled drug treatment groups using a two-tailed independent *t*-test, and Bonferroni-adjusted *p*-value was used to determine significance. NS (non-significance) is defined by *p* > 0.05.

## Supplementary Materials

The following are available online at https://www.mdpi.com/2227-9059/8/12/589/s1, Video S1: Voluntary feeding by Tg2576 mice in the oral vehicle and oral PIO groups.
